# A Computational Model of Quantitative Chromatin Immunoprecipitation (ChIP) Analysis

**DOI:** 10.4137/cin.s295

**Published:** 2008-03-12

**Authors:** Jingping Xie, Philip S. Crooke, Brett A. McKinney, Joel Soltman, Stephen J. Brandt

**Affiliations:** 1 Department of Medicine, Vanderbilt University, Nashville, Tennessee 37232, U.S.A; 2 Department of Cell and Developmental Biology, Vanderbilt University, Nashville, Tennessee 37232, U.S.A; 3 Department of Cancer Biology, Vanderbilt University, Nashville, Tennessee 37232, U.S.A; 4 Department of Mathematics, Vanderbilt University, Nashville, Tennessee 37232, U.S.A; 5 Department of Pediatrics, Vanderbilt University, Nashville, Tennessee 37232, U.S.A; 6 Vanderbilt-Ingram Cancer Center, Vanderbilt University, Nashville, Tennessee 37232, U.S.A; 7 VA Tennessee Valley Healthcare System, Nashville, Tennessee 37212, U.S.A; 8 University School of Nashville, Nashville, Tennessee 37212, U.S.A; 9 Current address: Department of Genetics, University of Alabama Birmingham Medical Center, Birmingham, Alabama 35294, U.S.A

**Keywords:** chromatin immunoprecipitation analysis, computer modelling, transcription factors

## Abstract

Chromatin immunoprecipitation (ChIP) analysis is widely used to identify the locations in genomes occupied by transcription factors (TFs). The approach involves chemical cross-linking of DNA with associated proteins, fragmentation of chromatin by sonication or enzymatic digestion, immunoprecipitation of the fragments containing the protein of interest, and then PCR or hybridization analysis to characterize and quantify the genomic sequences enriched. We developed a computational model of quantitative ChIP analysis to elucidate the factors contributing to the method’s resolution. The most important variables identified by the model were, in order of importance, the spacing of the PCR primers, the mean length of the chromatin fragments, and, unexpectedly, the type of fragment width distribution, with very small DNA fragments and smaller amplicons providing the best resolution of TF binding. One of the major predictions of the model was also validated experimentally.

## Introduction

Numerous cellular processes rely on the coordinate expression of large numbers of individual genes. This is orchestrated by transcription factors (TFs), sequence-specific DNA-binding proteins that activate or repress gene expression by affecting the assembly of RNA polymerase complexes. Thus, elucidation of the genomic sequences to which TFs bind is essential in order to understand their biological actions.

A variety of approaches have been used to discover the genes regulated by specific TFs. One of the first developed involves characterization of the mRNAs induced by the enforced expression of a TF in the presence of a protein synthesis inhibitor (Rosa, 1989). Although this method can identify genes whose transcription is affected by that TF, it cannot determine if or where the TF is bound.

In the event that a TF’s DNA-binding preference is known, a candidate gene approach can be used. This may be applied at the level of individual genes or genome-wide (Iyer et al. 2001; Lieb et al. 2001; Cawley et al. 2004) and the most likely sequences evaluated for protein-binding activity by electrophoretic mobility shift analysis (Garner and Revzin, 1981). Since TFs can interact with DNA *in vitro* but not be associated with the corresponding sequences in cells and can be recruited by other DNA-binding proteins to sequences that they would not otherwise bind, this approach cannot establish the validity of any target with certainty.

Both to overcome these difficulties and to assess DNA occupancy in a more physiologic context, the techniques of chromatin immunoprecipitation (ChIP) and Dam methylase identification (DamID) were developed. Besides confirming that a given gene is occupied by a specific TF *in vivo*, DamID (van Steensel et al. 2003) and ChIP (Cohen-Kaminsky et al. 1998; Orlando, 2000; Orian et al. 2003) analysis have been useful in target gene discovery, particularly when combined with promoter (Li et al. 2003), CpG island (Weinmann et al. 2002), or whole chromosome (Martone et al. 2003) arrays.

In all applications of the ChIP technique, TFs and other associated proteins are cross-linked to DNA *in situ*, chromatin is sheared or enzymatically digested, and the fragments containing the protein of interest are collected with an antibody. Enrichment of specific genomic sequences is then quantified by PCR or hybridization analysis. To define the variables that contribute to the assay’s resolution, we developed a computational model of quantitative ChIP analysis. Here we describe the results of simulations with this model and experimental studies validating one of its predictions.

## Results

### Computational model of ChIP analysis

A computational model of quantitative ChIP analysis (code available on request) was created with *Mathematica* (Wolfram Research, Champaign, IL). It assigned a single TF binding site 530 bp from the 5′ end of a 1.5 kb linear DNA molecule, subdivided the molecule mathematically, with the separation points determined by a probability distribution whose mean was the desired fragment length, and then queried the resulting fragments for the presence of the TF and two primer binding sites. Fragments containing all three sites were counted and the total divided by the number of DNA molecules, generally set at 50,000, to give a frequency for that fragment length and primer pair location. Finally, the location of the midpoint between the primers and relative frequency with which specific fragments were selected were compiled. These steps were repeated for a number of mean fragment lengths, primer locations, and fragment width distributions.

The computational model thus simulated formaldehyde cross-linking of a TF to its DNA binding site, mechanical shearing of chromatin by sonication, and immunoselection of the chromatin fragments containing the TF. For simplicity, it assumed complete identity of the assigned and actual TF binding sites and uniformity in protein-DNA cross-linking, primer affinities for DNA, and PCR reaction efficiencies.

In the first set of simulations, an exponential distribution of chromatin fragment lengths was assumed and a set of non-overlapping primers was spaced uniformly across the DNA. An exponential distribution was utilized by Kim and colleagues in their treatment of ChIP-on-chip analysis (Kim et al. 2005) and a rationale for its use based on the maximum entropy principle is developed here (see Supplemental Material).

The computational model was used to examine the factors important for assay resolution, in particular the lengths of the DNA fragments generated by sonication and the sizes of the DNA molecules (amplicons) dictated by the spacing of the PCR primers. Computer simulations were carried out for mean fragment lengths of 140, 150, 200, 250, 500, and 750 bp and amplicons of 50, 100, 150, 200, and 250 bp. A total of 50,000 DNA molecules were analyzed, which equates to a cell number of 25,000 for a diploid organism.

**Figure 1:**
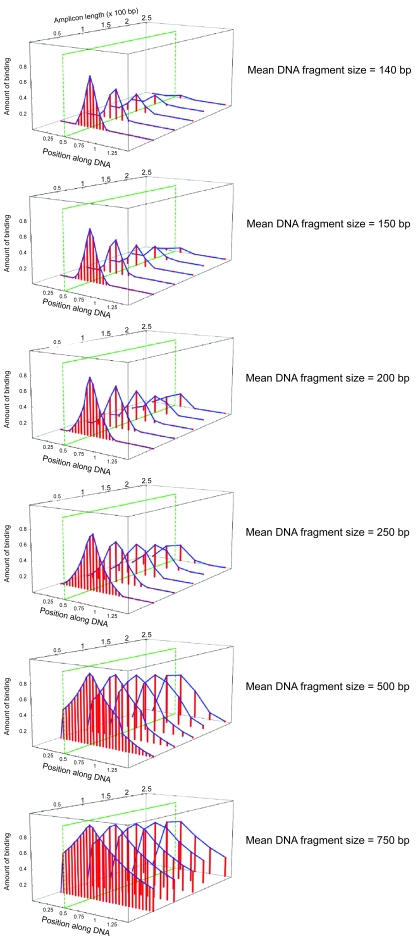
Results of computational simulation of quantitative ChIP analysis: exponential distribution TF binding, quantified using a scale from 0–1, is plotted as a function of position along a 1.5 kb linear DNA molecule. A single TF binding site (marked by *green line*) was assigned to a position 530 bp from its 5′ end. The amount of binding was determined for amplicons of 50, 100, 150, 200, and 250 bp and a mean DNA fragment size of 140–750 bp as indicated. An exponential distribution of fragment widths was used for these simulations.

As expected, the model showed that the location giving the greatest signal in the simulated PCR analysis coincided with the assigned TF binding site (Fig. 1). Although extent rather than probability of binding was analyzed, the width and inflection point of the resulting binding isotherm would determine the precision with which the TF binding site could be localized and represent two measures of assay resolution. As shown in Figure 1, the model shows that the shorter the distance between PCR primers for given sized DNA fragments, the more narrowly the binding distribution encompassed the TF binding site. With increasing amplicon size, the PCR signal declined, ultimately falling to zero when the size of the amplification product exceeded DNA fragment length. When the length of the amplicon was fixed and the DNA fragment size varied, the binding isotherm narrowed with decreasing mean DNA fragment size. Thus, the model shows that small DNA fragments and short amplicons provide the greatest precision in localizing TF binding, although the binding isotherms for many DNA fragment sizes and amplicon lengths possess distinct peaks (Fig. 1) and a number of combinations could potentially be informative.

While theoretical considerations support an exponential distribution (see Supplemental Material), there is no consensus on which distribution best describes the range of DNA fragment sizes that result from sonication of chromatin. Therefore, we also carried out simulations using the normal distribution, testing the variables of mean DNA fragment length, amplicon size, and standard deviation of mean fragment size (σ). As above, the simulations assumed a pool of 50,000 DNA molecules.

**Figure 2:**
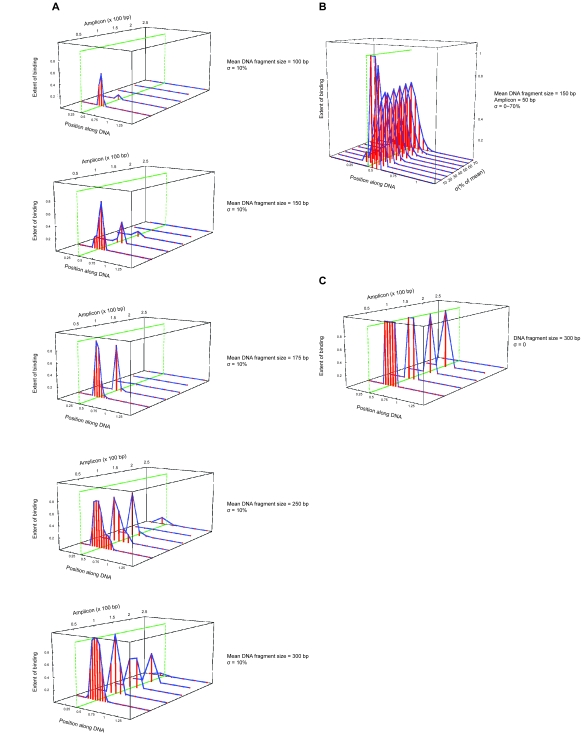
Results of computational simulation of quantitative ChIP analysis: normal distribution TF binding, quantified according to a scale from 0–1, is plotted as a function of position along a 1.5 kb linear DNA molecule. A single TF binding site (marked by *green line*) was assigned to a position 530 bp from its 5′ end. (**A**) Distribution of binding was determined for amplicons of 50, 100, 150, 200, and 250 bp, mean DNA fragment sizes of 100, 150, 175, 250, and 300 bp, and σ = 10% of mean. (**B**) Distribution of binding was determined for a mean DNA fragment size of 150 bp, amplicon of 50 bp, and σ = 0%–70% of mean. (**C**) Distribution of binding was determined for DNA fragments of identical size (300 bp, σ = 0) and amplicons of the indicated lengths. A normal distribution of fragment widths was used for these simulations.

Using a range of DNA fragment sizes and σ = 10%, the model reveals that the smaller the amplicon, the more closely the binding isotherm flanked the binding site. Unexpectedly, for certain fragment widths (e.g. 300 bp), resolution varied inconsistently with changes in amplicon size, and better resolution was obtained for this fragment width with amplicons of 100 and 250 bp than 50 and 150 bp (Fig. 2A). Overall, however, resolution increased and signal strength declined with decreasing fragment size. In contrast, resolution was not significantly affected by variation in fragment width, and little or no difference in the binding isotherm was observed over a range of values of σ (Fig. 2B). Finally, when all DNA fragments were the same length (i.e. σ = 0) and the primers closely spaced, the model produced a “mesa”-like binding curve (Fig. 2C), exactly as described by the simple model of Kadosh and Struhl (Kadosh and Struhl, 1998). Thus, the type of fragment width distribution can interact with the variables of mean DNA fragment length and amplicon size in affecting the shape of the binding isotherm. Nevertheless, the greatest resolution using a normal distribution of DNA fragment widths was achieved with the smallest sized DNA fragments (e.g. 100 bp) and amplicons (e.g. 50 bp), similar to the exponential distribution.

### Test of the computational model

**Figure 3:**
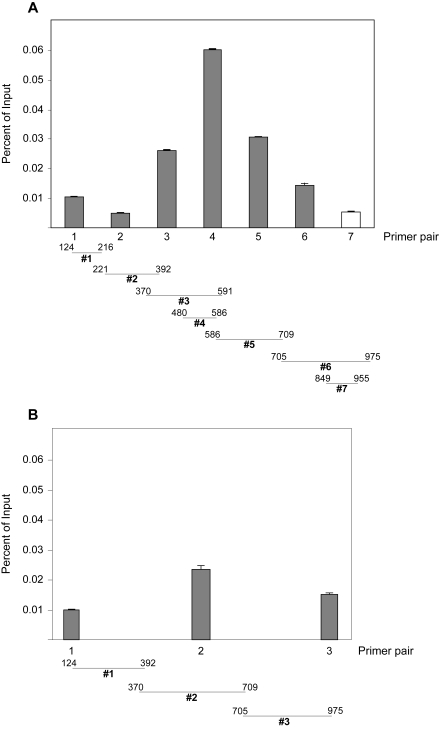
Results of quantitative ChIP analysis of upstream region of the mouse *Band 3* gene (**A**) Results of quantitative ChIP analysis of a region upstream of the *Band 3* gene, with the numbers denoting the relative position in this region. Data are expressed for each primer pair as a percentage of input DNA. Specific binding was determined by subtracting binding with rabbit IgG from that with affinity-purified rabbit Tal1 antibody. (**B**) Results of PCR analysis of the same collection of DNA fragments using primer pairs directing the amplification of longer DNA products. Data are expressed as a percentage of input DNA. The same scale is used for the ordinate in **A** and **B** so that the amount of TF binding could be compared directly.

To test the predictions of the computer model, quantitative ChIP analysis was carried out for a region upstream of the gene for the erythrocyte membrane protein Band 3. A series of primer pairs of different spacing was applied to a common set of DNA fragments, with some of the amplicons necessarily overlapping because of constraints in primer design. In preliminary studies, the helix-loop-helix TF Tal1 was found to occupy this region in murine erythroleukemia cells and was shown to be recruited by another TF, Runx1, to one or more of its five binding sites in the 106 bp interval defined by primer pair #4. Importantly, these primers also gave the greatest signal for Tal1 occupancy in quantitative ChIP analysis (Fig. 3A). Consistent with the computational model, shorter amplicons produced a binding isotherm more closely centered over the determined site(s) of Tal1 association (Fig. 3A), and signal strength diminished and the binding curve flattened with wider spacing between primers (compare Fig. 3B to Fig. 3A). Detailed analysis of the sizes of ethidium bromide-stained DNA fragments from this experiment showed that neither an exponential nor the normal distribution precisely fit the experimental data. Nevertheless, these studies bear out the major prediction of the model, that for a given mean DNA fragment size, the use of smaller amplicons would, in general, provide greater resolution than longer amplicons.

## Discussion

We developed a new computational model of ChIP analysis and used it to test the effects of chromatin fragment length and PCR primer spacing on assay resolution. When the TF binding site is not apparent from inspection of nucleotide sequence, as for the Tal1 target gene *Band 3* described here, it is a decided advantage to evaluate as short a stretch of DNA as possible for TF interaction. Although other models of the ChIP method have been described, they addressed different questions and focused on hybridization rather than PCR analysis of the immunoprecipitated DNA (Jin et al. 2006; Johnson et al. 2006; Qi et al. 2006).

Our model predicts that amplicons and sonicated DNA fragments of similar length would provide the best combination of resolution and signal strength. This was also the conclusion reached by a group applying a joint binding deconvolution method to ChIP-on-chip analysis (Qi et al. 2006). For maximal resolution, however, the model suggests use of very finely sheared chromatin and closely spaced primers.

The influence of reducing fragment length and amplicon size on the assay’s resolving ability was, unexpectedly, impacted by the type of fragment width distribution used in the computer simulations. Since there is no consensus as to which distribution best describes the sizes of DNA fragments that result from sonication, we tested both an exponential and the normal distribution. Although neither fit precisely the fragment width distribution that was observed experimentally and our model did not factor in any variability in protein cross-linking, primer affinity, or PCR efficiency, experimental and computational results both indicated that very small DNA fragments and smaller amplicons provide the most precise localization of TF binding.

## Methods

### Computational model

A 1.5 kb double-stranded DNA molecule containing a single TF binding site 530 bp from its 5′ end was assumed and computer simulations carried out on a total of 50,000 such molecules. Mean fragment length and PCR primer locations (spacing) were selected and the DNA molecule subdivided mathematically, with the separation points determined by a probability distribution whose mean was the desired fragment length (or any other positive number). A standard deviation of the distribution was also selected for two-parameter probability distributions (e.g. the normal distribution). Each fragment was then evaluated for the presence of the primer and TF binding sites and the number of fragments containing all three sites was counted. This number was divided by 50,000 to determine the relative frequency of that particular fragment length and primer pair location, and the location of the midpoint of the primer pair and relative frequency were recorded. These steps were repeated for different fragment lengths and primer pair locations. Computer code for the *Mathematica* program is available on request.

### Chromatin immunoprecipitation analysis

MEL cells (line F4-12B2) were cultured in Dulbecco’s Modified Eagle Medium (DMEM) with 10% fetal bovine serum, 100 units/ml penicillin and 100 μg/ml streptomycin. A total of 1 × 10^8^ cells at a density of ~1 × 10^6^ cells/ml were cross-linked with 1% formaldehyde for 10 minutes at 37 °C. Crosslinking was terminated by the addition of glycine at a final concentration of 100 mM. Cells were washed with phosphate-buffered saline and lysed with a hypotonic buffer (10 mM Tris-HCl, pH 7.5, 10 mM KCl, 1.5 mM MgCl_2_, 0.1% NP40, 1 mM DTT) containing a cocktail of protease inhibitors (Sigma-Aldrich, St. Louis, MO) including 0.5 mM phenylmethylsulfonyl fluoride. Nuclei were collected by centrifugation at 500 g for 5 min, resuspended in 4 ml of sonication buffer (10 mM Tris HCl, pH 8.0, 150 mM NaCl, 0.05% SDS, 0.5% Triton X-100, 0.5% NP-40, 0.1% sodium deoxycholate, 1 mM EDTA), and sheared with a probe sonicator (Virsonic 600, Virtis, Gardiner, NY). A mean DNA size of ~140–150 bp was achieved with nine 25 s pulses at a power setting of 3.5. Following sonication, chromatin was pre-cleared with 200 μl of Protein A-agarose and aliquots incubated with affinity-purified Tal1 antibody or normal rabbit IgG overnight at 4 °C. Subsequent washing and purification of DNA were carried out with reagents from Upstate Cell Signaling Solutions (Charlottesville, VA) according to the manufacturer’s recommendations. Precipitated DNA was resuspended in 100 μl of 0.1 × TE (10 mM Tris HCl, pH 8.0, 1 mM EDTA) and analyzed by real-time PCR. To quantify precipitation efficiency, DNA from the input control was diluted and amplified using the same procedure. PCR primers were designed with the aid of MacVector software (Accelrys, San Diego, CA), with the product size ranging from 75 to 350 bp. PCR analysis was carried out with reagents from Bio-Rad Laboratories (Hercules, CA) in a final volume of 25 μl using an initial denaturation for 3 min at 94 °C followed by 45 cycles of denaturation for 10 s at 94 °C and annealing and extension for 1 min at 65 °C. The sizes of PCR products were characterized by ethidium bromide staining of acrylamide gels and their identities confirmed by DNA sequencing.

## Description of Additional Data Files

### Additional file 1. Rationale for exponential distribution

Rationale for use of an exponential distribution in DNA fragment length based on the maximum entropy principle and a simple statistical argument (see document in Supplemental Material).

#### Rationale for Exponential Distribution

In this supplemental material, we justify the exponential fragment width in two ways: using the maximum entropy principle and through a simple statistical argument. First we derive a probability distribution for the fragment widths using the maximum entropy principle (Jaynes, 1957) assuming only the average amplicon width as prior information. The maximum entropy principle states that the least biased probability distribution *p**_i_* maximizes the information entropy

(A1) $$H=-\sum_{i=1}^{N}p_{i}\text{ln}p_{i}$$

under the constraints of prior information. For the sonication process, our only prior assumptions are that the probability field is normalized and the average width of the fragments is Λ:

(A2) $$\sum_{i=1}^{N}p_{i}=1$$

(A3) $$\sum_{i=1}^{N}p_{i}x_{i}=\Lambda$$

where *x**_i_* is the spectrum of fragment widths. To maximize the information entropy under these constraints, we introduce the Lagrange multipliers λ_1_′ and λ_2_′, and we require for all *k* that

(A4) $$\frac{\partial }{\partial p_{k}}\left(-\sum_{i=1}^{N}p_{i}\text{ln}p_{i}+\lambda_{1}^{\prime }\left(\sum_{i=1}^{N}p_{i}-1\right)+\lambda_{2}^{\prime }\left(\sum_{i=1}^{N}p_{i}x_{i}-\Lambda \right)\right)=0,$$

which leads to

(A5) $$p_{k}=e^{-\lambda_{1}}e^{-\lambda_{2}x_{k}},$$

where we have defined the new constants λ_1_ = 1 − λ_1_′ and λ_2_ = − λ_2_′. Constraint (A1) determines λ_1_ giving

(A6) $$p_{k}=\frac{e^{-\lambda_{2}x_{k}}}{Z},$$

where *Z* = ∑*_i_*_=1_*^N^**e*^−^*^λ^*^_2_^*^x_k_^* is the normalization constant. Constraint (A2) determines λ_2_:

(A7) $$\frac{1}{Z}\sum_{i=1}^{N}x_{i}e^{-\lambda_{2}x_{i}}=\Lambda .$$

The large number of fragments (*N* >>1) justifies approximating the sum as an integral so that Eq. (A7) becomes:

(A8) $$\frac{1}{Z}\int_{0}^{\infty }xe^{-\lambda_{2}x}dx=\Lambda ,\;\text{where }Z=\int_{0}^{\infty }e^{-\lambda_{2}x}dx,$$

which are easily integrated to give λ_2_ = 1/Λ. Finally, we obtain the least biased probability for the width of fragment *k* given only the average width as a constraint:

(A9) $$p_{k}=\frac{1}{\Lambda }e^{-x_{k}/\Lambda }.$$

An elegant property of the maximum entropy principle is that it allows one to obtain a fragment-width distribution without making any extraneous assumptions about the microscopic process of sonication. This approach leads to excellent agreement with the ChIP experiments, but for completeness we introduce a simple, physically motivated description of the sonication process that leads to the same fragment-width distribution. Essentially we assume that the shearing of a point along a DNA sequence is statistical in nature so that each basepair has the same probability of being cleaved. The probability of cleavage in a short sequence interval *dx* is δ · *dx*, where δ is a phenomenological parameter that has units of 1/length. The decrease *dN* in the number of fragments of width *x* in the sequence interval *dx* is proportional to the number of fragments *N* of width *x* and the cleavage probability δ · *dx*:

(A10) dN=-Nδ·dx,

which integrates to give

(A11) $$N=N_{o}e^{-\delta \cdot x},$$

where *N**_o_* can be interpreted as the number of fragments with zero width. However, we simply treat *N**_o_* as a normalization constant so that *N**_o_* = δ and Eq. (A2) becomes a fragment-width probability distribution:

(A12) $$P(x)=\delta \cdot e^{-\delta \cdot x}.$$

Using Eq. (A12) as our probability distribution for the number of fragments with width between *x* and *x* + *dx*, we can calculate the average width, which we equate with the experimentally observed value Λ:

$$\Lambda =\int_{0}^{\infty }xP(x)dx=\delta \int_{0}^{\infty }xe^{-\delta \cdot x}dx,$$

which gives δ = 1/Λ, and Eq. (A12) is, thus, equivalent to Eq. (A9).
